# A measure of SRS/SRT plan quality: Quantitative limits for intermediate dose spill (R50%) in linac‐based delivery

**DOI:** 10.1002/acm2.13570

**Published:** 2022-03-02

**Authors:** Dharmin D. Desai, Ivan L. Cordrey, E. L. Johnson

**Affiliations:** ^1^ Department of Radiation Oncology CHI Memorial Hospital Chattanooga Tennessee; ^2^ Regional Cancer Center Cumberland Medical Center Crossville Tennessee; ^3^ Department of Radiation Medicine University of Kentucky Chandler Medical Center Lexington Kentucky

**Keywords:** intermediate dose spill, LowerR50%, R50%, R50% range, SRS, SRT, UpperR50%

## Abstract

Stereotactic radiosurgery (SRS) and stereotactic radiotherapy (SRT) of multiple cranial targets using a single isocenter on conventional C‐arm linear accelerators are rapidly developing clinical techniques. However, no universal guidelines for acceptable intermediate dose spill limits are currently available or widely accepted. In this work, we propose an intermediate dose spill guidance range for cranial SRS/SRT delivered on C‐arm linacs with MLC collimation for single PTV plans and single isocenter multiple target plans with PTV volumes in the range 0.02–57.9 cm^3^. We quantify intermediate dose spill with the R50% metric (R50% = volume of 50% of prescription isodose cloud / volume of PTV) and test the proposed range using three clinical data sets, containing both 6 MV and 10 MV beams, previously published by other authors. Our proposed lower limit of R50% (LowerR50%) and upper limit of acceptable R50% (UpperR50%) bound over 90% of the clinical data used in this study, yet still provide a challenging benchmark for optimization and plan assessment of linac‐based, MLC collimated SRS/SRT.

## 1 INTRODUCTION

Stereotactic radiosurgery (SRS) and stereotactic radiotherapy (SRT) aim to deliver high doses of ionizing radiation to cranial targets in one or a few fractions with high precision. The application of linac‐based SRS/SRT in the treatment of brain metastases has become increasingly common in radiation therapy clinics; however, the cranium contains many organs at risk (OARs) requiring consideration in plan optimization, such as the brainstem, optic chiasm, and optic nerves. Dose threshold recommendations for these OARs are specified in AAPM Task Group 101 (TG‐101).^1^

Minimizing normal brain tissue dose is also an important optimization and planning objective because normal brain is an OAR always directly adjacent to planning target volume (PTV) surfaces and subject to the higher doses being delivered to these PTVs. Indeed, radiation necrosis of normal brain tissue is one of the more relevant adverse effects after SRS/SRT.^2^ Brain radionecrosis has been correlated with the volume of brain that receives a dose of 12 Gy (V12Gy) for single fraction SRS and V18Gy for multi fraction SRT.^3^, ^4^

Intermediate dose spill is often tracked and reported in SRS/SRT studies by various metrics. The computation of these metrics often utilizes the volume that receives at least 50% of the prescription dose (Rx), which we refer to as *V*_IDC50%_. Because isodose lines are concentrically nested and SRS/SRT prescriptions are often between 16 and 24 Gy, the *V*_IDC50%_ is often close to V12Gy or V18Gy.^5^ Thus, *V*_IDC50%_ and intermediate dose spill metrics derived from it are reasonable surrogates for normal brain radionecrosis. Yet, at this time, there is limited published guidance regarding acceptable intermediate dose spill for cranial SRS/SRT.

In contrast, for lung stereotactic body radiotherapy (SBRT), the Radiation Therapy Oncology Group (RTOG) protocols 0813 and 0915 provide early consensus guidelines for acceptable intermediate dose spill in terms of the intermediate dose spill metric, R50%.^6^, ^7^

(1) $$R50\%=\frac{V_{IDC50\%}}{V_{PTV}},$$

where *V*_IDC50%_ is the volume of the 50% Rx isodose cloud and *V*_PTV_ is the volume of the PTV. As has been commented in another publication, no comparable guidelines exist for cranial SRS/SRT at this time.^8^

When considering volumetric modulated arc therapy (VMAT) SRS/SRT, and especially single isocenter multiple target VMAT, the lack of an accepted range for intermediate dose spill presents an opportunity for improvement. Several authors have offered clinical data studies that include data fits that provide some guidance based on an individual institution's experience. Yet, what has been achieved in the past does not explicitly reveal what can be achieved with improved optimization strategies, especially when one considers what the best achievable plan could be.

For SRS/SRT cranial irradiation, we propose guidelines for a range of intermediate dose spill quantified by R50% that would define a plan with R50% as low as reasonably achievable. To test the proposed range for R50%, we conduct a semiempirical assessment of three published SRS/SRT data sets.

## 2 METHODS

### 2.1 Proposed R50% limits

Desai et al. derived a semiempirical expression for R50% based on a spherical PTV model; they called this expression R50%_Analytic_.^9^ R50%_Analytic_ is dependent on the volume of the PTV (*V*_PTV_), surface area of the PTV (*SA*_PTV_), and a distance of dose drop‐off to 50% parameter (∆*r*). The parameter ∆*r* must be determined empirically. They compared R50%_Analytic_ to another author's previously published SRS clinical data set and demonstrated that R50%_Analytic_ was a reasonable approximate lower bound for what could be achieved in single target SRS/SRT delivered on a C‐arm linear accelerator using MLC collimation.^9^ The expression for R50%_Analytic_ is given below.

(2) $$\begin{array}{c} R50{\%}_{Analytic}=1+\frac{SA_{PTV}}{V_{PTV}}\Delta r\left[1+\left(\frac{\Delta r}{r_{PTV}}\right)+\frac{1}{3}{\left(\frac{\Delta r}{r_{PTV}}\right)}^{2}\right], \end{array}$$

where *r*_PTV_ is the effective sphere PTV radius given by

(3) $$r_{PTV}={\left(\frac{3}{4\;\pi }V_{PTV}\right)}^{1/3},$$

and the ∆r is given by^9^

(4) $$\Delta r\;=\;0.2844\;{\left(V_{PTV}\right)}^{0.1973}$$

for which *V*_PTV_ is in cm^3^ and the resulting ∆*r* values are in cm.

R50%_Analytic_ has been previously suggested as a planning objective for single isocenter multiple target SRS/SRT by Desai et al.^10^

Since a sphere is the minimum surface area solid volume, the minimum value of R50%_Analytic_ for any given PTV volume would be for a spherical PTV. We propose a lower bound of R50% values, LowerR50%, that would be R50%_Analytic‐Sphere_ for spherical PTVs. Substituting the equations for surface area and volume of a sphere into Equation  (2) yields

(5) $$\begin{array}{ccc} & & LowerR50\%\;=\;R50{\%}_{Analytic-Sphere\;}\\ & & \;=1+\frac{3}{r_{PTV}}\Delta r\left[1+\left(\frac{\Delta r}{r_{PTV}}\right)+\frac{1}{3}{\left(\frac{\Delta r}{r_{PTV}}\right)}^{2}\right] \end{array}$$

with ∆*r* and *r*_PTV_ given above (Equations 3 and 4).

If LowerR50% constitutes a reasonable approximate lower bound for R50%, the next relevant step is to determine what constitutes a reasonable upper bound for R50% values, UpperR50%. Assessing a large data set published by Popple et al.,^11^ we phenomenologically determined an upper bound that encompassed approximately 90% of the autoplanned HyperArc data that had R50% < 16. The HyperArc data were shown superior to manual planned data^11^ and are taken here as representative of a best‐case scenario for clinical plans treated with 10 MV flattening filter free beams (10X‐FFF). This proposed upper limit for R50% is given as

(6) $$UpperR50\%\;=\;1.1\;+\;5.4\;{\left(V_{PTV}\right)}^{-0.35}.$$

Therefore, we propose a range of R50% values specific for linac‐based MLC collimated SRS/SRT for which the lower bound is LowerR50% (Equation 5), and the upper bound is UpperR50% (Equation 6).

### 2.2 Published data comparison

We assess three previously published data sets representing both single target and single isocenter multiple target plans with which to compare the proposed R50% limits. The reader is referred to the specific articles for the planning details that generated these data sets.

Data published by Zhao et al. (Zhao data set) is used to assess the proposed R50% limits against 6 MV beam (6X) single target SRS/SRT. The data provided by Zhao et al. were for single targets planned with 6X beams using dynamic conformal arc therapy (DCAT) delivery.^5^ This represents a carefully curated data set of plans retrospectively optimized to minimize intermediate dose spill as quantified by the gradient index (GI). This data was converted to R50% by the relationship

(7) R50%=CI×GI.

The details of the individual PTVs are published directly in the article of Zhao et al., and as such, we have direct access to specific datapoints for 30 PTVs with an effective diameter in the range 6.6–45.8 mm.^5^

Aggregate data published by Ballangrud et al.^12^ are used to assess the proposed R50% limits against 6X single isocenter multiple target SRS/SRT. Ballangrud et al. published a data fit equation (Ballangrud data fit) that roughly averaged their clinical data such that roughly 50% of the data fell above the fit and 50% fell below the data fit.^12^ As shown by Desai et al., the clinical data fit of Ballangrud et al. for GI can be converted to an equivalent R50% by multiplying their GI expression by the reported average Conformity index (CI).^10^ The Ballangrud et al. data are represented by the curve given by

(8) $$R50{\%}_{Ballangrud}\;=\;4.8\;{\left(V_{PTV}\right)}^{-0.2}.$$

Some institutions use 10X‐FFF beams for linac‐based, MLC collimated VMAT SRS/SRT delivery. Popple et al. at the University of Alabama Birmingham (UAB) published an extensive database of clinical SRS/SRT plans using 10X‐FFF VMAT delivery (UAB data set).^11^ Their results showed that their autoplanning methods yielded lower R50% results on average than cases planned by manual methods. This comprehensive data set was provided to us in tabulated form via private communication and is taken as representative of well‐optimized plans for single target and single isocenter multiple target plans. The data published describes intermediate dose spill with a metric they call Falloff Index, which is mathematically equivalent to R50% (Equation 1).^11^ We focus on the 713 PTVs with an effective diameter in the range 3.24–48 mm and R50% < 16.

We include each data set on a plot of R50% versus target diameter along with the LowerR50% and UpperR50% for assessment of the proposed limits.

## 3 RESULTS

**FIGURE 1:**
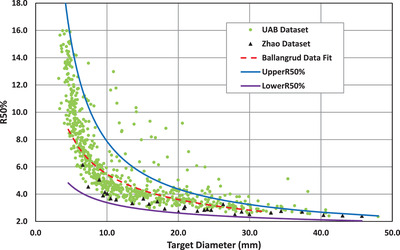
Comparison of proposed R50% limits for linac‐based MLC collimated SRS/SRT (LowerR50% and UpperR50%) with previously published data. The light green circles represent the UAB data set that includes both single and single isocenter multiple target cases treated with 10X‐FFF beams and VMAT delivery with R50% <16.^11^ The black triangles represent the Zhao data set that includes single target cases treated with 6X beams for dynamic conformal arc delivery.^5^ The dashed red line represents the clinical Ballangrud data fit (Equation 8) that averages their multiple target VMAT cases.^12^ The UpperR50% (Equation 6), shown as the blue line, is designed to capture 90% of the UAB data set. The LowerR50% (Equation 5), shown as the purple line, is clearly a lower bound for the three clinical data sets

Comparison results are summarized in Figure 1, which shows the three data sets on a plot presented with the proposed LowerR50% and UpperR50%. Notice that most of the clinical data are between the proposed optimal R50% limits.

The light green circles represent the UAB data set that includes both single and single isocenter multiple target cases treated with 10X‐FFF beams and VMAT delivery with R50% < 16.^11^ The black triangles represent the Zhao data set that includes single target cases treated with 6X beams for dynamic conformal arc delivery.^5^ The dashed red line represents the clinical Ballangrud data fit (Equation 8) that averages their multiple target VMAT cases.^12^ The UpperR50% (Equation 6) is shown as the blue line. The LowerR50% (Equation 5), shown as the purple line, is clearly a lower bound for the three clinical data sets.

The R50% graph is plotted as a function of the target diameter instead of the target volume because of the data density of the UAB data set for small targets. The plot as a function of target diameter provides a cubic stretch of the horizontal axis small volume targets (where there is a very high data density) and a corresponding compression of the large volume region of the graph where the data is sparser.

## 4 DISCUSSION

From Figure 1, we can clearly see that the LowerR50% (Equation 6) is a reasonable lower bound to all the data presented. The UAB data set approaches the LowerR50% in several instances, as does the Zhao data set, but the planned data R50% values are never less than the LowerR50%.

The UpperR50% is an upper bound of 90% of the UAB data set shown by design, so the phenomenological fit holds true for the entire spectrum data up to 48 mm PTV effective diameter. It can be difficult to fully resolve in the figure shown, but the UpperR50% is slightly larger than the 48 mm UAB data point.

The data fit equation representing the data of Ballangrud et al. appears to fall conveniently halfway between the UpperR50% and Lower50% across the entire spectrum of effective PTV diameter.

The three data sets shown represent a range of current linac‐based, MLC collimated SRS/SRT: single target DCAT with 6X beams, single isocenter multiple target VMAT with 6X beams, single target VMAT with 10X‐FFF beams, and single isocenter multiple target with 10X‐FFF. This is not a comprehensive review of all available data since many published data sets are not readily available, but it is a reasonable sample of data generally recognized as representing well‐optimized plans for linac‐based cranial SRS/SRT.

The span in R50% between the LowerR50% and UpperR50% is not uniform from the small targets to the large targets. This is actually an important aspect of these proposed limits. The range needs to be wider for small targets because a small change in *V*_IDC50%_ for a small V_PTV_ can manifest a very large change in R50%. For example, take *V*_PTV_ = 0.1 cm^3^ with an R50% = 7.7; an increase in R50% to 11.7 (increase of 4) represents a change in *V*_IDC50%_ of 0.4 cm^3^ (as calculated from Equation 1) of additional nontarget brain receiving insult of the 50%+ dose. It is debatable whether this difference has detectable clinical impact. Thus, the broader span between the LowerR50% and UpperR50% for small PTVs represents the clinically acceptable variation based on relevant impact to nontarget brain.

The span between the LowerR50% and UpperR50% needs to be considerably smaller for large *V*_PTV_ because a small change in R50% results in a much larger change in the *V*_IDC50%_ at the large end of the PTV volume range. For example, take *V*_PTV_ = 58 cm^3^ with R50% = 2.13; an increase in R50% to 2.53 (an increase of only 0.4) represents 23.2 cm^3^ of additional nontarget brain receiving the 50%+ dose, which many would agree has clinical implications for the patient. Fortunately, the proposed LowerR50% and UpperR50% reflect the clinical needs at both ends of the PTV size spectrum.

The LowerR50% is a very important benchmark of plan quality. Regardless of the chosen optimization strategy, one should pursue treatment plans that come as close to LowerR50% as possible. This LowerR50% is more likely approachable for single target plans than for the R50% of an individual PTV in a single isocenter multiple target plan because of MLC limitations in the leaf sequence that leave leaf pairs bridging the gap between two PTV with an open leaf gap that irradiates the nontarget brain tissue between the PTVs, as well as other complicating factors (MLC leakage, beam geometry, etc.). However, knowing where the LowerR50% is for a given V_PTV_ allows the planner to push the optimizer toward the optimum solution instead of settling for an inferior optimization.

The LowerR50% is unlikely to change meaningfully for linac‐based, 6 MV MLC collimated SRS/SRT as more data is accumulated, but the UpperR50% may be decreased as optimization techniques improve and more data becomes available. Yet, for now, there should be some standard that defines an acceptable plan; thus, we propose these R50% limits for consideration by the medical physics community as an initial benchmark.

A convenient advantage of the proposed limits is that the limits are not tabulated values but rather computable mathematical formulas. As such, they are easy to create within scripted planning systems or programmed into autoplanning routines, even artificial intelligence implementations.

The proposed R50% range in this study is agnostic to the optimization technique and beam geometry. Any optimization technique that achieves highly conformal SRS/SRT plans can be tested against the proposed R50% limits. The proposed R50% range for SRS/SRT gives treatment planners guidelines to strive for regardless of their chosen planning techniques.

## 5 CONCLUSION

We propose a R50% guidance range for cranial SRS/SRT delivered on C‐arm linacs with MLC collimation that appears sound for single PTV plans and single isocenter multiple target plans with well‐separated PTV volumes in the range 0.02–57.9 cm^3^ (3.6–48 mm effective PTV diameter). The lower limit of the range is the semiempirical approximate minimum given by LowerR50% (Equation 5); the upper limit of the range is UpperR50% (Equation 6). The clinical data provided by the Zhao and UAB data sets and the Ballangrud data fit provide evidence that the proposed R50% range is reasonably achievable. We propose this R50% range for testing and evaluation at other facilities.

## CONFLICT OF INTEREST

No conflicts of interest.

## AUTHOR CONTRIBUTIONS

All authors contributed equally to this project.

## FUNDING STATEMENT

There are no funders to report for this submission.

## Data Availability

The data that support the findings of this study are available from the corresponding author upon reasonable request.
